# A new relation between prevalence and incidence of a chronic disease

**DOI:** 10.1093/imammb/dqu024

**Published:** 2015-01-09

**Authors:** Ralph Brinks, Sandra Landwehr

**Affiliations:** German Diabetes Centre, Institute for Biometry and Epidemiology, Auf’m Hennekamp 65, 40225 Duesseldorf, Germany

**Keywords:** incidence, prevalence, mortality, compartment models, chronic diseases, dementia

## Abstract

In 1991 Keiding published a relation between the age-specific prevalence and incidence of a chronic disease (in Age-specific incidence and prevalence: a statistical perspective. *J. Roy. Stat. Soc. A*, **154**, 371–412). For special cases alternative formulations by differential equations were given recently in Brinks *et al.* (2013, Deriving age-specific incidence from prevalence with an ordinary differential equation. *Statist. Med.*, **32**, 2070–2078) and in Brinks & Landwehr (2014, Age- and time-dependent model of the prevalence of non-communicable diseases and application to dementia in Germany, *Theor. Popul. Biol.*, **92**, 62–68). From these works, we generalize formulations and discuss the advantages of the novel approach. As an implication, we obtain a new way of estimating the incidence rate of a chronic disease from prevalence data. This enables us to employ cross-sectional studies where otherwise expensive and lengthy follow-up studies are needed. This article illustrates and validates the novel method in a simulation study about dementia in Germany.

## 1. Introduction

One of the objectives of epidemiology is the description of health-related states and events in populations. To achieve this objective, incidence and prevalence are important quantitative concepts. Incidence refers to the occurrence of new cases in a specific health-related state during a time period, whereas prevalence measures the proportion of subjects who are in the state at a point in time. Both measures are fundamental in epidemiological research.

For analysing quantitative aspects of infectious diseases, state models (synonymously: compartment models) are widely used and have a history going back at least to the 1920s (see, for example, Brauer, 2005). With respect to chronic diseases, compartment models are less common and have appeared later (Fix & Neyman, 1951). The infrequent use of mathematical models in this field is in contrast to the tremendous worldwide burden of chronic diseases. For example, two-thirds of all global cases of death in 2010 have been attributed to chronic diseases (Lozano, 2012). Hence, we feel the urgent need to contribute to the mathematical understanding of the worldwide epidemics of chronic diseases.

**Fig. 1.**
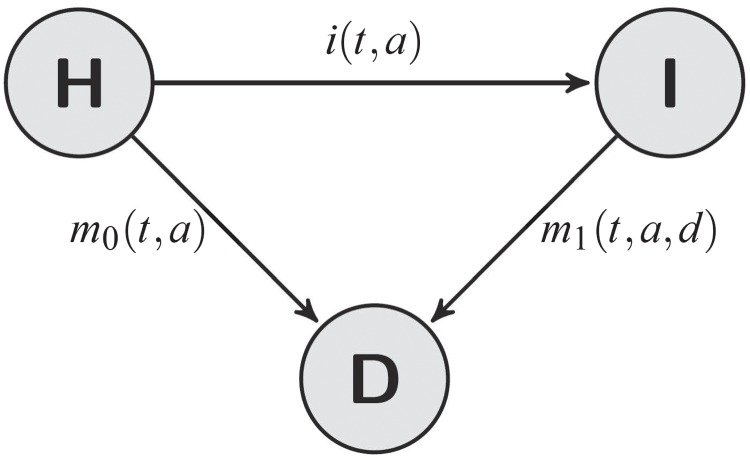
Compartment model with three states and transition rates depending on different time scales: calendar time $t,$ age $a$ and duration $d.$

A typical model in the epidemiology of chronic diseases considers a population in three states: healthy (H), diseased (I) and dead (D) (Keiding, 1991). Subjects of the population may undergo irreversible transitions between these states as shown in Fig. 1. The transition rates are the incidence $i,$ and the mortalities $m_0$ and $m_1$ of the healthy and the diseased subjects, respectively.

In many situations, it is important to keep track of different time scales (Keiding, 2006). Mortality, for instance, crucially depends on the age of the subjects, but also on secular progress in hygiene, nutrition and medical care. Hence, the rates $i$ and $m_0$ may depend on age $a$ and on calendar time $t$. In addition, the rate $m_1$ may also depend on the duration $d$ of the disease (Fig. 1).

In the literature, two approaches can be found in dealing with the state model and its transition rates. Keiding (1991) chose a stochastic nomenclature, whereas the group around Murray & Lopez preferred differential equations (Murray & Lopez, 1994, 1996; Barendregt *et al.*, 2003). According to the long tradition of differential equations in modelling infectious diseases, we follow the way of differential equations. We show that our approach is able to obtain Keiding's result (Keiding, 1991).

## 2. Methods

Let $S(t, a)$ denote the absolute number of subjects aged $a$ at time $t$ in state H. Moreover, let $C(t, a, d)$ be the number of people age $a$ at time $t$ who are in state I for exact duration $d.$ The total number of subjects aged $a$ at $t$ who have the chronic disease is $C^\star (t, a) = \int _0^a C(t, a, \delta ) \, \mathrm {d}\delta .$

In this article, we made three assumptions:

1. The population is closed, i.e. there is no migration.
2. We consider only diseases contracted after birth. Thus, it holds $C^\star (t, 0) = 0$ for all $t.$
3. The functions $S$ and $C$ are sufficiently smooth.

### 2.1. Keiding's equation

If we look at the change rates of the subjects in the states, balance equations for $S$ and $C$ can be formulated as follows:

(2.1) \[(\partial _t + \partial _a) S(t, a) = -(m_0(t, a) +i(t, a)) S(t, a)\]

(2.2) \[(\partial _t + \partial _a + \partial _d) C(t,a,d) = - m_1(t, a, d) C(t, a, d).\]

For ease of notation we have written $\partial _x = {\partial }/{\partial x}$ for $x \in \{ t, a, d\} .$ Equations (2.1–2.2) are partial differential equations (PDEs) that describe the outflows from the states H and I, respectively. The first of these equations implies that leaving the state H is a *competing risk* of the events *Death without having contracted the disease* and *Contracting the disease* (Putter *et al.*, 2007). The system of PDEs (2.1–2.2) is extended by the following initial conditions:

\[\begin {align} S(t - a, 0) & = S_0(t - a), \\ C(t,a, 0) & = i(t, a)S(t, a). \end {align}\]

The first initial condition describes the number of (disease-free) newborns, and the second describes the number of newly diseased persons at $(t, a)$.

The PDEs with the initial conditions have the following solutions:

(2.3) \[S(t, a) = S_0(t - a) \exp \left ( - \int _0^a m_0(t-a+ \tau , \tau ) +i(t-a+ \tau , \tau ) \, \mathrm {d}\tau \right ) .\]

\[\begin {align}C(t, a, d) &= C(t-d, a-d, 0) \exp \left ( - \int _0^d m_1(t-d+ \tau , a-d+ \tau , \tau ) \, \mathrm {d}\tau \right ) \\ &= i(t-d, a-d) S(t-d, a-d) \exp \left ( - \int _0^d m_1(t-d+ \tau , a-d+ \tau , \tau ) \, \mathrm {d}\tau \right ) .\end {align}\]

For brevity we define

\[M_1(t, a, d):= \int _0^d m_1(t-d+ \tau , a-d+ \tau , \tau ) \, \mathrm {d}\tau .\]

Then, the total number $C^\star$ of diseased subjects is

(2.4) \[C^\star (t, a) = \int _0^a i(t-\delta , a-\delta ) S(t-\delta , a-\delta ) \exp (-M_1(t, a, \delta ))\, \mathrm {d}\delta\]

By inserting Equations (2.3) and (2.4) into the definition of the age-specific prevalence

\[p(t, a) = \frac {C^\star (t, a)}{S(t, a) +C^\star (t, a)}\]

we get the following theorem.

**Theorem 2.1 (Keiding, 1991)**

*The prevalence* $p(t, a)$ *of those aged* $a \ge 0$ *at time* $t$ *can be calculated by*

(2.5) \[p(t, a) = \frac {\int \nolimits _0^a i(t-\delta , a-\delta ) \mathcal {M}_{t,a}(a-\delta ) \exp (- M_1(t, a, \delta ))\,\mathrm {d}\delta } {\mathcal {M}_{t,a}(a) + \int \nolimits _0^a i(t-\delta , a-\delta ) \mathcal {M}_{t,a}(a-\delta ) \exp (- M_1(t, a, \delta ))\,\mathrm {d}\delta },\]

*with*

\[\mathcal {M}_{t, a}(y):= \exp \left ( -\int _0^y m_0(t-a+ \tau , \tau ) +i(t-a+ \tau , \tau ) \mathrm {d}\tau \right ) .\]

Given the incidence rate $i$ and the mortality rates $m_k, ~k = 0, 1,$ Equation (2.5) analytically describes the prevalence $p$ of the chronic disease for a specific age $a$ and at a specific point in time $t$. The formula reflects the complex interplay of the involved incidence and mortality rates.

Unfortunately, Equation (2.5) is rarely used in epidemiology or public health contexts. One of the reasons may be that only a few researchers are aware of the equation and its potential. A huge advantage of the equation is the possibility of simulating scenarios. For instance, in the context of planning future health resources one might ask: What would be the effect of reducing the incidence of a specific chronic disease by 25% on the prevalence in the age group 60–80? What would be the effect of lowering the mortality $m_1$ of the diseased persons by 10%?

These are important questions in predicting the effects (e.g. outcomes, costs, budget impact etc.) of interventions or health programmes. Thus, we think the equation can contribute in planning the allocation of health resources or in the field of health policy decision-making.

### 2.2. Partial differential equations

In this section we formulate another relation between prevalence and incidence. We start with a lemma.

**Lemma 2.1**

*The total number* $C^\star$ *of diseased persons aged* $a \ge 0$ *at* $t,$$C^\star (t, a) = \int _0^a C(t, a, \delta ) \, \mathrm {d}\delta ,$ *is the solution of the initial value problem*

\[\begin {align} (\partial _t + \partial _a) C^\star (t, a) &= - m^\star _1(t, a) C^\star (t, a) +i(t, a) S(t, a). \\ C^\star (t-a, 0) = 0 \end {align}\]

*with*

(2.6) \[m^\star _1(t, a):=\left \{ \begin {array}{ll}\dfrac {\int \nolimits _0^a m_1(t, a, \delta ) C(t, a, \delta )\,\mathrm {d}\delta } {\int \nolimits _0^a C(t, a, \delta )\,\mathrm {d}\delta } & \textrm {for } C^\star (t, a) >0\\ 0 & \textrm {for } C^\star (t, a) = 0. \end {array}\right .\]

**Proof**

May be found in Appendix.

With the lemma we are able to derive the main result of this article.

**Theorem 2.2**

*The age-specific prevalence* $p$ *is the solution of the initial value problem*

(2.7) \[(\partial _t + \partial _a) p = (1-p)(i - p (m^\star _1 - m_0)),\]

*with* $p(t, 0) = 0.$

**Proof**

By applying the quotient rule to $p = \tfrac {C^\star }{S +C^\star }$ and substituting the expressions for $(\partial _t + \partial _a) S$ and $(\partial _t + \partial _a) C^\star$ we get Equation (2.7).

Before we describe the advantages of Equation (2.7), we show that it is a generalization of the relations found in Brinks *et al.* (2013) and Brinks & Landwehr (2014). If $m_1$ is independent from $d,$ i.e. $m_1(t, a, d) = m_1(t, a),$ then it holds $m_1 = m^\star _1$ and (2.7) becomes

(2.8) \[(\partial _t + \partial _a) p = (1-p) (i - p (m_1 - m_0)),\]

which has been shown in Brinks & Landwehr (2014). If in addition all rates are independent from $t$, one obtains the ordinary differential equation as in Brinks *et al.* (2013). Hence, Equation (2.7) is an extension of our previously published results if the mortality $m_1$ of the diseased depends on the duration $d$. For some chronic diseases, there is epidemiological evidence that $d$ plays a crucial role for $m_1$, for example, in diabetes (Carstensen *et al.*, 2008) and systemic lupus erythematosus (Bernatsky *et al.*, 2006).

Compared with Keiding's Equation (2.5) the PDE approach is simpler and has a greater flexibility, which is illustrated in three points. The first point is a new possibility of estimating incidence rates from prevalence data. This is an important application in epidemiology and is demonstrated in the next section. The second advantage of the PDE approach becomes obvious, when the information about the mortality is not given in terms of the mortality rates $m_0$ and $m_1$ of the healthy and the diseased population, respectively, but in terms of the *general mortality* $m$ of the whole population and the relative mortality $R = {m^\star _1}/{m_0}.$ While Keiding's Equation (2.5) is not able to calculate the prevalence $p$ in this situation, the PDE is. A brief calculation using the relation $m = pm^\star _1 + (1-p)m_0$ shows that $p$ is the solution of the PDE

(2.9) \[(\partial _t + \partial _a) p=(1-p)\left ( i-m \frac {p(R - 1)}{p(R - 1) +1} \right ) .\]

The situation of given $m$ and $R$ is very common in epidemiology and public health. Often, the general mortality $m$ can be obtained from official vital statistics or life tables. The relative mortality $R$ is taken from disease-specific surveys. Then, Equation (2.9) is able to calculate the prevalence whereas Keiding's formula is not. An anonymous reviewer gave us the valuable hint that Brunet & Struchiner (1999) also derived a relation between prevalence odds ${p}/{(1-p)}$, incidence $i$ and mortalities $m_k,\ k=0, 1,$ in terms of a PDE, which is similar to Equation (2.8). Similar to Keiding's formula, the approach of Brunet & Struchiner (1999) is not able to cope with the situation when $m$ and $R$ are given instead of $m_0$ and $m_1.$

**Remark 2.1**

The fraction on the right-hand side in (2.9) is the *population attributable fraction*, a well-known epidemiological quantity (Kirkwood & Sterne, 2003).

Finally, the greater flexibility of the PDE compared with Keiding's and Brunet and Struchiner's formula is apparent if we release the assumption of a closed population. Keiding and Brunet & Struchiner do not cover this case, whereas by an extension of the PDE (2.7) this is easily possible. The necessary steps are described in Brinks & Landwehr (2014).

**Remark 2.2**

Equation (2.7) uses calendar time $t$ and age $a$ as underlying (independent) variables and describes the change of the prevalence as a function of $t$ and $a$. This may seen in the light of the celebrated *McKendrick–Von Foerster Equation*, which does the same for the population density (in a closed population). For a review of the history and further references, see the excellent overview by Keiding (2011).

### 2.3. Estimation of the age-specific incidence from two cross-sectional studies

The primary advantage of the PDE approach over Keiding's Equation (2.5) is a possibility of deriving incidence rates from prevalence data. We start with the observation that in contrast to (2.5), the PDE (2.7) can be solved for the incidence rate $i:$

(2.10) \[i = \frac {(\partial _t + \partial _a) p}{1-p} +p (m^\star _1 - m_0).\]

This equation provides a way to estimate the age-specific incidence from two cross-sectional studies. Consider two points in time, $t_0$ and $t_0 + \Delta ,\ \Delta >0,$ and assume we know the age-specific mortalities $m_0(\cdot , a)$ and $m^\star _1(\cdot , a)$ at calendar time $t_0 + {\Delta }/{2}.$ Then, Equation (2.10) is the basis for the following algorithm:

**Algorithm 2.1 (Incidence from two cross-sections)**

Let the age-specific prevalence $p(\cdot , a)$ be given at $t_0$ and $t_0 + \Delta ,\ \Delta >0.$ Set $\tilde t = t_0 + {\Delta }/{2}.$

1. Approximate $p(\tilde t, a)$ by
  (2.11) \[p(\tilde t, a) \doteq \frac {1}{2}\left [ p\left ( t_0 + \Delta , a + \frac {\Delta }{2}\right ) +p\left ( t_0, a - \frac {\Delta }{2}\right ) \right ] .\]
2. Similarly, approximate $(\partial _t + \partial _a) p$ at $(\tilde t, a)$ by
  (2.12) \[(\partial _t + \partial _a) p (\tilde t, a) \doteq \frac {1}{\Delta } \left [ p\left ( t_0 + \Delta , a + \frac {\Delta }{2}\right ) - p\left ( t_0, a - \frac {\Delta }{2}\right ) \right ] .\]
3. Estimate the age-specific incidence by Equation (2.10):
  \[i(\tilde t, a) = \frac {(\partial _t + \partial _a) p (\tilde t, a)}{1-p(\tilde t, a)} +p (\tilde t, a) (m^\star _1 (\tilde t, a) - m_0 (\tilde t, a)).\]

While the last step in Algorithm 2.1 is mathematically exact, the algorithm comprises two approximation steps (indicated by the ‘$\doteq$’ sign), which are sources for errors. First, an error occurs for approximating the prevalence $p(\tilde t, a)$ by the mean of $p(t_0, a - {\Delta }/{2})$ and $p(t_0 + \Delta , a + {\Delta }/{2})$ in Equation (2.11). The second error arises in estimating the partial derivative $(\partial _t + \partial _a) p$ by the finite difference in (2.12).

In both approximations, the underlying idea is *linearization*, i.e. the assumption that the intermediate value in (2.11) and that the derivative in (2.12) can be approximated by linear functions. If the prevalence $p$ was a linear function, both steps would yield the associated exact values and the errors would be equal to zero. In practical applications, one would not choose the time lag $\Delta$ between the two cross-sections too long [but long enough to gain a reliable estimate in (2.11) and (2.12)].

## 3. Example

For illustration of the practical relevance, we apply the theory to an example motivated by dementia in German males. The mortality $m_0$ of the non-diseased is chosen to be

\[m_0(t, a) = \exp (-9.0 +0.085a - t \log _e (1.01)),\]

which is an approximation of the age-specific mortality of the male German population aged $\ge 50$ in the past six decades (Federal Statistical Office of Germany, 2011). The calendar time $t$ is given in years since 1960.

The age-specific incidence of dementia is assumed to be

(3.1) \[i(t, a) = i(a) = \exp (-12.8 +0.11 a), \quad a \ge 50.\]

This is an approximation of the observed rate in males (Ziegler & Doblhammer, 2009). As there are indications that the age-specific incidence is relatively stable (Qiu *et al.*, 2009), we consider it to be independent from calendar time $t.$

Concerning the mortality $m_1$ of the men with dementia, we examine two cases: $m_1$ being *independent* and being *dependent* on the disease duration $d.$ In both cases, we use Keiding's Equation (2.5) to calculate the age-specific prevalence of dementia in the years 2010 and 2015. This mimics two cross-sectional studies with a time lag of 5 years ($\Delta = 5$). The two cross-sections are used to derive the age-specific incidence rate in at $t = 2012.5$ by Algorithm 2.1. As we know the true incidence underlying the simulation, we can compare the estimates of Algorithm 2.1 with the true values given by (3.1). In this way, we compare our estimate with our own input and do not need additional data for validation.

### 3.1. Independence from duration

In the first example, we assume that the mortality $m_1$ of the diseased is independent from the duration $d.$ Even more, $m_1$ is considered proportional to $m_0:$ $m_1(t, a, d) = m^\star _1(t, a) = R \ m_0(t, a).$ The *relative mortality* $R (={m_1}/{m_0})$ is chosen to be $R = 2.63,$ which is the average value of the relative mortality in the first 6 years after diagnosis of dementia in a comparable English population (Rait *et al.*, 2010).

**Fig. 2.**
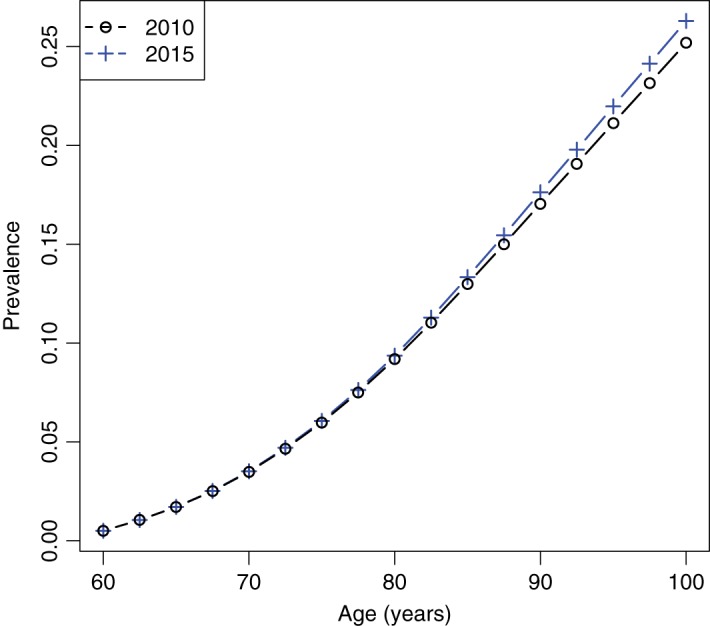
Age-specific prevalence in 2010 and 2015 (example *without* duration dependency).

The age courses of the prevalence in 2010 and 2015 are calculated by Keiding's Equation (2.5) in steps of 2.5 years length $a = 60, 62.5, \ldots , 97.5, 100.$ The integrals have been calculated using Romberg's method, which allows a prescribed accuracy (Dahlquist & Björck, 1974). The results are shown in Fig. 2.

Based on the age course of the prevalence in Fig. 2, we apply Algorithm 2.1 with $m^\star _1 (t, a) = 2.63 \ m_0 (t, a).$ The results are shown in Table 1.

**Table 1.** Comparison between the true and the calculated age-specific incidence rates in the first example.

Age $a$	True incidence	Calculated incidence	Relative error (%)
62.5	0.0026718	0.0027006	1.08
65.0	0.0035175	0.0035542	1.04
67.5	0.0046309	0.0046861	1.19
70.0	0.0060967	0.0061226	0.42
72.5	0.0080266	0.0080156	$-$0.14
75.0	0.0105672	0.0105659	$-$0.01
77.5	0.0139120	0.0138738	$-$0.28
80.0	0.0183156	0.0181919	$-$0.68
82.5	0.0241131	0.0238324	$-$1.16
85.0	0.0317456	0.0312618	$-$1.52
87.5	0.0417941	0.0411196	$-$1.61
90.0	0.0550232	0.0540997	$-$1.68
92.5	0.0724398	0.0712442	$-$1.65
95.0	0.0953692	0.0938397	$-$1.60
97.5	0.1255564	0.1238085	$-$1.39

Comparing the true and the calculated incidence rates, we see that the absolute value of the relative error for all ages $a = 62.5, \ldots , 97.5$ is less than 2%.

### 3.2. Duration dependency

The second example mimics the mortality $m_1$ being dependent on the duration since onset of the disease. According to the values reported in the study of Rait *et al.* , we model

\[m_1(t, a, d) = R(d) \ m_0(t, a).\]

Again, we calculate the age-specific prevalence in the years 2010 and 2015 using Keiding's Equation (2.5). The resulting age-specific prevalence is similar to the prevalence shown in Fig. 2.

If we want to extract the age-specific incidence as in the previous section, we should know $m_1^\star .$ Although $m_1^\star$ may be accessible by epidemiological surveys, in our setting we do not know the exact rate, because the distribution ${C(t, a, d)}/{\int _0^a C(t, a, \delta ) \mathrm {d}\delta }$ in Equation (2.6) is unknown. We present two ways to overcome this problem in practice: (a) we apply Algorithm 2.1 as in the previous section with setting $m^\star _1 (t, a) = m_1(t, a) = 2.63 \ m_0 (t, a).$ The value 2.63 is the average of all the reported relative mortalities from year 1 to year 6 after diagnosis. (b) In the study by Rait *et al.* it has been observed that the persons aged $>90$ die quite soon after diagnosis of dementia. Thus, we set $m^\star _1 (t, a) = 2.755 \ m_0 (t, a)$ for $a >90$, where 2.755 is the average relative mortality from year 1 to year 4 after diagnosis. The comparisons of the estimated incidence rates with the true values are shown in Table 2. The third and fourth columns refer to method (a) and the fifth and sixth columns refer to method (b).

**Table 2.** Comparison between the true and the calculated age-specific incidence rates in the second example.

Age $a$	True incidence	Calc. inc.$^{{\rm a}}$	Rel. error$^{{\rm a}}$ (%)	Calc. inc.$^{{\rm b}}$	Rel. error$^{{\rm b}}$ (%)
62.5	0.0026718	0.0026940	0.83	0.0026940	0.83
65.0	0.0035175	0.0035196	0.06	0.0035196	0.06
67.5	0.0046309	0.0046868	1.21	0.0046868	1.21
70.0	0.0060967	0.0062091	1.84	0.0062091	1.84
72.5	0.0080266	0.0081738	1.84	0.0081738	1.84
75.0	0.0105672	0.0107598	1.82	0.0107598	1.82
77.5	0.0139120	0.0141145	1.46	0.0141145	1.46
80.0	0.0183156	0.0186430	1.79	0.0186430	1.79
82.5	0.0241131	0.0244683	1.47	0.0244683	1.47
85.0	0.0317456	0.0318820	0.43	0.0318820	0.43
87.5	0.0417941	0.0414960	$-$0.71	0.0414960	$-$0.71
90.0	0.0550232	0.0537769	$-$2.27	0.0537769	$-$2.27
92.5	0.0724398	0.0693771	$-$4.23	0.0739852	2.13
95.0	0.0953692	0.0889364	$-$6.75	0.0951201	$-$0.26
97.5	0.1255564	0.1134120	$-$9.67	0.1215714	$-$3.17

$^{a}$Assumed relative mortality $2.63$. $^{b}$Assumed relative mortality $2.63$ for $a \le 90$ and $2.755$ for $a >90.$

Although the relation $m_1(t, a, d) = R(d) \ m_0(t, a)$ that has been used to generate the input data has not been utilized in Algorithm 2.1, the estimated age-specific incidence rates deviate only slightly for $a \lt 90,$ namely less than 2% in absolute terms. For ages 90 and more, the deviations increase with age in method (a), which has given rise to increase the relative mortality of this age group in method (b). The rationale behind method (b) is that in the age group $\ge 90$ only a small percentage survive $\ge 5$ years after diagnosis. Thus, averaging the relative mortality over years 1 to 6 gives too much weight on the later years after diagnosis, when the relative mortality is lower than in the early years after diagnosis.

## 4. Summary

In this article we have formulated and proven a new relation between the age-specific prevalence, the incidence and the mortality rates in terms of a PDE. The relation generalizes differential equations published recently in Brinks *et al.* (2013) and Brinks & Landwehr (2014). Compared with the relations from Keiding (1991) and Brunet & Struchiner (1999), the PDE is simpler and has a greater flexibility. The flexibility has been illustrated in three points: (i) a new way of deriving incidence rates from prevalence data, (ii) the use of the method if the general mortality is given instead of the mortality rates of the healthy and diseased and (iii) the possible extension in case of migration. A fourth aspect may be mentioned if we allow a transition from the disease state (I) back to the state (H). Again the PDE is capable to deal with this situation and Keiding is not, see Brinks & Landwehr (2014) for details.

The new method of deriving incidence rates from prevalence data may be very useful in epidemiology. While prevalence data may be obtained by cross-sectional studies, the estimation of incidence rates mostly require lengthy and costly follow-up studies. Especially in low or middle income countries data about incidence of many diseases have not been surveyed yet. Furthermore, in some situations, estimates from cross-sectional data might be more reliable than estimates by follow-up studies. For example, in surveying occurrence of health states where subjects might feel uncomfortable or even stigmatized, losses to follow-up or withdrawals of consent are very likely. An anonymous cross-section may be found more acceptable and less intrusive for study participants than repetitive re-examinations.

With a view to practical applications of Algorithm 2.1, apart from the approximation errors, sampling errors in surveying the age-specific prevalence have to be considered. The sampling error depends on several parameters and a discussion is beyond the scope of this article. For an introduction about this issue we refer to Brinks *et al.* (2013) and the associated technical appendix, where sampling error was assessed in simulation studies. Error bounds arising from uncertainties in raw population data may be obtained by bootstrap methods as described and demonstrated in Brinks *et al.* (2013).

In summary, we have presented a new relation between the age-specific prevalence, the incidence and the mortality rates. The relation is applicable in many contexts from epidemiology, public health and demography. Furthermore, it is simpler and more flexible than a previously found equation. With our findings, we hope to contribute to the quantitative understanding of how basic epidemiological rates and processes may impact global health and burden of chronic diseases.

## Appendix: Proof of Lemma 2.1

We have to show that $C^\star$ is the solution of the PDE $(\partial _t + \partial _a) C^\star = - m_1^\star C^\star +iS$. With $\partial = \partial _t + \partial _a$ it holds:

\[\begin {align} \partial C^\star (t, a) &= \partial \int \nolimits _0^a C(t, a, \delta ) \, \mathrm {d}\delta \\ &= \int \nolimits _0^a \partial C(t, a, \delta ) \, \mathrm {d}\delta +C(t, a, a)\\ &= \int \nolimits _0^a (\partial _t + \partial _a + \partial _d) C(t, a, \delta ) \, \mathrm {d}\delta - \int \nolimits _0^a \partial _d C(t, a, \delta ) \, \mathrm {d}\delta +C(t, a, a)\\ &= - \int \nolimits _0^a m_1(t, a, \delta ) \, C(t, a, \delta ) \, \mathrm {d}\delta - \int \nolimits _0^a \partial _d C(t, a, \delta ) \, \mathrm {d}\delta +C(t, a, a)\\ &= - m_1^\star (t, a) C^\star (t, a) - \bigl (C(t, a, a) - C(t, a, 0) \bigr ) +C(t, a, a) \\ &= - m_1^\star (t, a) C^\star (t, a) +i(t, a) \, S(t, a). \end {align}\]

For the second equality Leibniz's integral rule has been used.
